# Diabetes, gender, and left ventricular structure in African-Americans: the atherosclerosis risk in communities study

**DOI:** 10.1186/1476-7120-4-43

**Published:** 2006-11-08

**Authors:** Murilo Foppa, Bruce B Duncan, Donna K Arnett, Emelia J Benjamin, Philip R Liebson, Teri A Manolio, Thomas N Skelton

**Affiliations:** 1 Graduate Studies Program in Cardiology, School of Medicine, Federal University of Rio Grande do Sul, Porto Alegre, Brazil; 2 Department of Epidemiology, University of North Carolina, Chapel Hill, NC, USA; 3 Division of Epidemiology and Community Health, School of Public Health, University of Minnesota, Minneapolis, MN, USA; 4 Evans Department of Medicine, Boston University School of Medicine, Boston, MA, USA; 5 Section of Cardiology, Rush Medical College, Rush University Medical Center, Chicago, IL, USA; 6 Division of Epidemiology and Clinical Applications, National Heart, Lung, and Blood Institute, Bethesda, MD, USA; 7 University of Mississippi Medical Center, MI, USA

## Abstract

**Background:**

Cardiovascular risk associated with diabetes may be partially attributed to left ventricular structural abnormalities. However, the relations between left ventricular structure and diabetes have not been extensively studied in African-Americans.

**Methods:**

We studied 514 male and 965 female African-Americans 51 to 70 years old, in whom echocardiographic left ventricular mass measurements were collected for the ARIC Study. In these, we investigated the independent association of diabetes with left ventricular structural abnormalities.

**Results:**

Diabetes, hypertension and obesity prevalences were 22%, 57% and 45%, respectively. Unindexed left ventricular mass was higher with diabetes in both men (238.3 ± 79.4 g vs. 213.7 ± 58.6 g; p < 0.001) and women (206.4 ± 61.5 g vs. 176.9 ± 50.1 g; p < 0.001), respectively. Prevalence of height-indexed left ventricular hypertrophy was higher in women while increased relative wall thickness was similar in men and women. Those with diabetes had higher prevalences of height-indexed left ventricular hypertrophy (52% vs. 32%; p < 0.001), and of increased relative wall thickness (73% vs. 64%; p = 0.002). Gender-adjusted associations of diabetes with left ventricular hypertrophy (OR = 2.29 95%CI:1.79–2.94) were attenuated after multiple adjustments in logistic regression (OR = 1.50 95%CI:1.12–2.00). Diabetes was associated with higher left ventricle diameter (OR = 2.13 95%CI:1.28–3.53) only in men and with higher wall thickness (OR = 1.89 95%CI:1.34–2.66) only in women. Attenuations in diabetes associations were frequently seen after adjustment for obesity indices.

**Conclusion:**

In African-Americans, diabetes is associated with left ventricular hypertrophy and, with different patterns of left ventricular structural abnormalities between genders. Attenuation seen in adjusted associations suggests that the higher frequency of structural abnormalities seen in diabetes may be due to factors other than hyperglycemia.

## Background

Left ventricular hypertrophy (LVH) is frequent in diabetic patients [1,2]. and has been identified as a powerful marker of impaired prognosis in cardiovascular disease [3], including in African-Americans [4].

Alterations in left ventricular (LV) structure have been linked to diabetes but also to a large number of related conditions such as aging, hypertension, obesity, central obesity, dyslipidemia, salt intake, and physical inactivity [5-7] Hence, the underlying processes common to the coexistent risk factors, as opposed to hyperinsulinemia or hyperglycemia, *per se*, may explain much of the association seen between diabetes and LV structural abnormalities.

African-Americans have a high prevalence of left ventricular structural abnormalities [8] and of its clinical correlates, frequently presented together [9,10] However, few population-based studies have adequately studied the relations between diabetes and these structural adaptive responses in African-Americans [11], which may have distinct pathophysiologic mechanisms [12], and also may differ between genders [13].

This study evaluates the independent cross-sectional associations of diabetes with LV structural alterations in a community-based sample of African-Americans with a high prevalence of other known risk factors for LVH.

## Methods

The ARIC Study is a prospective study designed to investigate the etiology and natural history of CVD in four U.S. communities. The study design and procedures, including the echocardiography protocol, have been previously reported [14,15]. Between 1987 and 1989, ARIC enrolled individuals 45 to 64 years old, and requested participants to return for clinical visits every three years through 1998. The initial Jackson (MI) center cohort consisted of 3728 African-Americans. Of these, echocardiography studies were performed in 863 men and 1571 women between 1993 and 1996. We excluded those with one or more of the following criteria: inadequate M-mode measurements (n = 549), clinically (n = 62) or echocardiographically (n = 212) diagnosed prevalent cardiovascular disease, and missing covariates (n = 132), leaving 1479 participants for analysis.

Diabetes was defined as a participant report of physician diagnosis or diabetic medication use, fasting glucose ≥ 126 mg/dL, or casual glucose ≥ 200 mg/dL. Hypertension was defined as a mean of two separate measures greater than 140/90 mmHg or antihypertensive drug use. Obesity was defined as a body mass index ≥ 30 kg/m^2^. Body surface area (BSA) was calculated as BSA = 0.007184 * weight [kg]^0.425 ^* height [cm]^0.725^, according to the Dubois formula [16]. Waist-hip ratio was calculated from measurements of waist (measured at the umbilicus) and hip (measured at maximum gluteal protrusion) circumferences.

The two-dimensional guided M-mode echocardiographic tracings were acquired with an Acuson 128XP/10c System. Images were digitized and analyzed off-line with a commercially available program. Only frames with optimal visualization of interfaces that simultaneously showed the interventricular septum, LV internal diameter, and posterior wall were used for readings. Tracings were read by one of two cardiologists, unaware of subject's clinical data, and the average value from 3 measurements was computed. LV mass was calculated using M-mode tracings according to American Society of Echocardiography conventions [17] and Devereux modified cubed formula [18]. To account for gender and body size variations we indexed LV mass employing height^2.7^, with a boundary of 51 g/m^2.7 ^to define LVH in both genders [19]. The intra- and intersonographer correlations for LV mass between the first and second scan, examined in a subsample, were 0.94 and 0.82, respectively. The intrareader correlation for LV mass was 0.98 [20].

Alterations in LV geometry were assessed by several approaches. Relative wall thickness (RWT) was calculated as the sum of wall thicknesses (septum + diastolic posterior wall thickness) divided by LV diastolic diameter, with elevated RWT defined as greater or equal to 0.45 [21]; In multivariate analysis increased LV diameter was defined as the upper quartile of gender specific left ventricular diameter. To define increased wall thickness for modeling, we used the median as cut-off point for the sum of wall thicknesses, as about two-thirds of both men and women in the sample had a RWT above 0.45.

We investigated the differences in crude prevalences between groups in stratified analyses with Chi-square statistics. We used logistic regression analysis (SAS 8.0 Software, Cary, NC) to study the independent association of variables with pre-defined echocardiographic features. Associations were adjusted for adiposity by including obesity, defined by BMI, and waist-hip ratio in models. For further, in multiple adjustment, we added the covariates age, systolic blood pressure, antihypertensive medication use, total cholesterol, activity level, smoking and education level. Height was also included as a covariate in analyses of increased LV diameter and increased wall thickness. In these multivariable models, we used clinically defined cut-points, when present, for continuous exposure variables. In order to produce exposure frequencies similar to that of diabetes, we modeled variables without pre-defined cut-points comparing the upper quartile to the lower three. Gender interactions were tested using the Wald statistic of the gender interaction term. We employed the variance inflation factor to identify collinearity among variables measuring potentially similar biological effects. The variance inflation factor was never greater than 3.0, suggesting that important collinearity was absent [22].

## Results

The 1479 individuals included in the study differed somewhat from the 955 subjects undergoing the exam but excluded from these analyses. The included participants had a similar gender distribution, somewhat lower prevalences of diabetes (22% vs. 25%; P = 0.22) and obesity (45% vs. 49%; P = 0.07), and less hypertension (57% vs. 64%; P = 0.02) than those excluded.

Of those studied, 514 (35%) were men and 965 (65%) were women. Compared with men, women had considerably higher prevalences of diabetes, obesity and hypertension (Table 1). Unindexed LV mass was higher with diabetes in both men (238.3 ± 79.4 g vs. 213.7 ± 58.6 g; p < 0.001) and women (206.4 ± 61.5 g vs. 176.9 ± 50.1 g; p < 0.001), as was height-indexed LV mass (men:52.4 ± 18.1 g/m^2.7 ^vs. 46.5 ± 13.2 g/m^2.7^, p < 0.01; women:55.2 ± 17.0 g/m^2.7 ^vs. 47.2 ± 13.5 g/m^2.7^, p = 0.001).

**Table 1:** Participant characteristics by gender

	Men (N = 514)	Women (N = 965)
Age – years	58.4 (5.8)	58.6 (5.6)
Diabetes (%)	98 (19%)	232 (24%)^†^
Obesity (%)	152 (30%)	509 (53%)^§^
Hypertension (%)	275 (53%)	576 (60%)^†^
Height – m	1.76 (0.07)	1.63 (0.06)^§^
Body Mass Index – kg/m^2^	28.1 (4.8)	31.2 (6.2)^§^
Systolic Blood Pressure – mmHg	130 (20)	131 (20)
Glucose – mg/dL	120 (57)	122 (56)
Smokers (%)	139 (27%)	145 (15%)^§^
Metabolic Syndrome^|| ^(%)	185 (36%)	501 (52%)^§^

Expressed as N (%) or mean (standard deviation);

P values testing the difference between genders: ^† ^<0.05; ^‡ ^<0.01; ^§ ^<0.001;

^|| ^Adapted from NCEP criteria [37].

LVH was a common finding, but the prevalence and sex differences in LVH varied depending on the indexation criteria used (Table 2). Compared with men, women had a higher prevalence of unindexed LVH, and of height indexed, but not BSA indexed LVH. Prevalence of increased RWT was similar in men and women.

**Table 2:** Echocardiographic measurements and prevalences of structural abnormalities in men and women*

	Men (N = 514)	Women (N = 965)
**Echocardiographic Measurements**		
Left Ventricular Diameter Diastole – mm	47.6 (5.7)	45.1 (5.4)^§^
Septum Thickness – mm	11.9 (2.5)	11.2 (2.2)^§^
Posterior Wall Thickness – mm	12.0 (2.2)	11.2 (1.9)^§^
Relative Wall Thickness (RWT)	0.51 (0.13)	0.50 (0.11)
Fractional Shortening (%)	37.0 (7.3)	40. (7.5)^§^
**Left Ventricular Mass Estimates||**		
LV mass – g	218.4 (63.8)	184.0 (54.6)^§^
LV mass/BSA – g/m^2^	107.2 (29.7)	97.3 (25.7)^§^
LV mass/Height^2.7 ^– g/m^2.7^	47.6 (14.4)	49.1 (14.8)^†^
**Structural Abnormalities**		
Unindexed LVH (38)	59 (11.5%)	339 (35.1%)^§^
Height^2.7 ^– Indexed LVH	169 (32.9%)	373 (38.6%)^†^
Relative Wall Thickness >= 0.45	332 (64.6%)	645 (66.8%)

*Expressed as N (%) or mean (standard deviation);

P values testing the difference between genders: ^† ^<0.05; ^‡ ^<0.01; ^§ ^<0.001;

^||^According to Devereux Modified Formula;

LV = Left Ventricular; BSA = Body Surface Area; LVH = LV Hypertrophy

**Figure 1:**
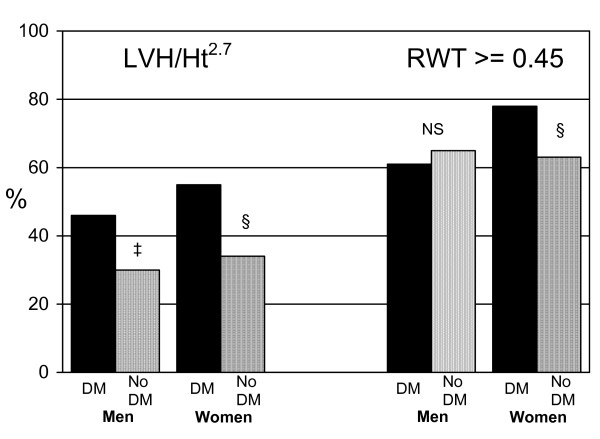
**Prevalences of structural abnormalities**. Prevalences of left ventricular hypertrophy and increased relative wall thickness in African-Americans with diabetes (black columns) and without diabetes (grey columns); P values diabetes vs. no diabetes: ^† ^<0.05; ^‡ ^<0.01; ^§ ^<0.001; NS non significant.

The unadjusted height-indexed LVH prevalences were higher in those with diabetes (52% vs. 32%; p < 0.001), obesity (55% vs. 22%; p < 0.001), and hypertension (48% vs. 21%; p < 0.001). Prevalences of increased RWT were also greater in those with diabetes (73% vs. 64%; P = 0.002) and hypertension (70% vs. 60%; P < 0.001), but not so in those classified as obese (68% vs. 64%; P = 0.07). However, sex-specific analyses showed that differences in prevalences of height-adjusted LVH associated with diabetes were similar in men and women, but the higher prevalence of increased RWT with diabetes was seen only in women (Figure 1; P < 0.01).

In sex-adjusted analyses, those with diabetes had a more than two-fold greater frequency of LVH (Table 3). Adjusting also for body mass index and waist-hip ratio reduced the magnitude of this association. Multiple adjustment, including also other relevant covariates, as described in the table footnotes, caused only a small further attenuation of this association (Table 3). Of note, in these models, is the relatively small size of the diabetes association, in comparison, in the same model, with that of obesity (OR = 3.63 95%CI 2.80–4.72), and the upper quartiles of waist-hip ratio (1.55 95%CI 1.17–2.06) and of systolic blood pressure (OR = 3.41 95%CI 2.59–4.48). Multiply-adjusted gender-specific associations were roughly similar in men (OR = 1.41 95%CI 0.83–2.41) and in women (OR = 1.50 95%CI 1.05–2.14).

**Table 3:** Crude and adjusted associations of diabetes with height-indexed left ventricular hypertrophy (LVH^2.7^)

	OR (95%CI)
*Unadjusted*	
Diabetes	2.29 (1.78–2.94)
Adiposity Adjusted*	
Diabetes	1.60 (1.22–2.10)
*Multiply Adjusted*^†^	
Diabetes	1.49 (1.12–2.00)
Gender (Male)	0.95 (0.72–1.26)
Obesity (BMI)	3.63 (2.80–4.72)
Waist-Hip Ratio^‡^	1.55 (1.17–2.06)
Systolic BP^‡^	3.41 (2.59–4.48)

* Model adjusted for gender, BMI and waist-hip ratio;

^† ^Model adjusted for gender, BMI, waist-hip ratio, systolic blood pressure, age, total cholesterol, activity level, smoking, education level and antihypertensive medication use;

^‡ ^4^th ^Quartile vs. rest of sample;

The unadjusted association of diabetes with relative wall thickness differed notably between men (OR = 0.84 95%CI 0.53–1.32) and women (OR = 2.06 95%CI 1.46–2.91; interaction P = 0.002). Multiply-adjusted gender-specific analyses revealed that diabetes was associated with increased RWT in women (OR = 1.63 95%CI 1.12–2.37) but not in men (OR = 0.64 95%CI 0.39–1.04).

Gender differences in RWT response were additionally investigated evaluating the RWT components – increased LV diameter and sum of wall thicknesses (Table 4). Multiply-adjusted gender-specific analysis showed that, in women, diabetes was not associated with larger LV chamber diameter (OR = 1.02 95%CI 0.70–1.48), but was with wall thickness (OR = 1.88 95%CI 1.34–2.65). In contradistinction, diabetes was significantly associated with larger LV chamber size (OR = 2.13 95%CI 1.28–3.53), in men, but not so with wall thickness (OR = 1.33 95%CI 0.81–2.18). In women, greater BMI was associated with both greater LV diameter (4^th ^quartile vs. the rest, OR = 3.43 95%CI 2.42–4.87) and wall thickness (OR = 1.68 95%CI 1.27–2.23); in men, with greater wall thickness (OR = 2.58 95%CI 1.63–4.07).

**Table 4:** Crude and adjusted gender-specific associations of diabetes with increased left ventricular diameter and wall thickness

	**Left Ventricular Diameter**		**Wall Thickness**	
	Men	Women	Men	Women

	OR (95%CI)	OR (95%CI)	OR (95%CI)	OR (95%CI)

*Unadjusted*				
Diabetes	2.36 (1.48–3.77)	1.31 (0.94–1.82)	1.77 (1.13–2.79)	2.70 (1.97–3.68)
Body-size Adjusted*				
Diabetes	2.10 (1.30–3.41)	1.01 (0.71–1.44)	1.48 (0.93–2.37)	2.04 (1.47–2.84)
*Multiply Adjusted*^†^				
Diabetes	2.13 (1.28–3.53)	1.02 (0.70–1.49)	1.33 (0.81–2.18)	1.89 (1.34–2.66)
BMI^‡^	1.22 (0.75–2.00)	3.43 (2.42–4.87)	2.58 (1.63–4.07)	1.68 (1.27–2.23)
Waist-Hip Ratio^‡^	1.50 (0.90–2.50)	0.88 (0.61–1.27)	1.09 (0.68–1.76)	1.85 (1.32–2.58)
Systolic BP^‡^	1.85 (1.16–2.96)	2.04 (1.44–2.89)	2.24 (1.43–3.52)	2.00 (1.44–2.79)

* Models adjusted for BMI, waist-hip ratio and height;

^† ^Models adjusted for BMI, waist-hip ratio, systolic blood pressure, height, age, total cholesterol, activity level, smoking, education level and antihypertensive medication use;

^‡ ^4^th ^Quartile vs. rest of sample;

## Discussion

Consistent with previous reports [8,9], LV structural abnormalities were frequent in this community sample of African-Americans. The prevalence of left ventricular hypertrophy was particularly high in women, even after body size adjustments. Within this context, we found an association of diabetes with LVH which, though diminishing with adjustment for obesity indices, was independent of body size. The association of LV structural abnormalities with diabetes [2], as well as with impaired glucose tolerance [23] in our African American cohort, is consistent with previous studies in predominantly or exclusively white cohorts.

Employment of relative wall thickness to evaluate geometric remodeling resulted in an unanticipated finding. Whereas RWT prevalences were similar between men and women, diabetes was associated with increased RWT in women; in men, if anything, with the reverse. Separate evaluation of the components of relative wall thickness – ventricular chamber size and wall thickness – produced a possible explanation for the apparent paradox: In women diabetes was associated with increased wall thickness, but not with increased left ventricular diameter. In men, on the contrary, diabetes was more strongly associated with increased left ventricular diameter than with increased wall thickness. This finding demonstrates a potential limitation of using the ratio of these two measures to identify the structural changes related to diabetes.

Differences in correlates of left ventricular structure may indicate that the pathophysiologic mechanisms leading to heart maladaptive response differ somewhat between genders. Females have been found to have an increased wall thickness response to pressure overload compared with males in clinical [13] and in animal studies [24]. These gender-specific differences in the impact of carbohydrate metabolism has also been identified in patients with the metabolic syndrome [25], but has been scarcely investigated in African-Americans. In non-diabetic American Indians of the Strong Heart Study [26] fasting insulin levels were associated with LV mass in men and with wall thickness in women. In the Framingham Study cohorts [1], increased LV mass and RWT were seen in diabetic women but not diabetic men. More recent analysis from the Framingham Heart Study [27], showed an increase in RWT parallel to diabetes severity and to insulin resistance (homeostasis model, HOMA-IR) only in women, although most of this association appeared to be explained by obesity. It is important to note that adjustment for indices of adiposity appeared to account for most of the reduction in the magnitude of diabetes associations seen in multiply-adjusted models. This finding was suggested by Galvan et al.[28], and is consistent with a recent review that concluded that associations of insulin and glucose metabolism with LV mass were mostly explained by adiposity [29]. In fact, one of the largest studies to date, the HyperGEN Study, with adequate representation of African-Americans, found inverse associations with fasting insulin levels and adaptive LV responses, once adjustment for several elements of the insulin resistance syndrome was performed [30]. Moreover, another analysis from the ARIC African-American participants showed greater interventricular and septal wall thicknesses in the obese, independent of blood pressure levels and diabetes [10].

The traditional explanation for the important association of obesity with abnormal LV responses is that obesity produces a hemodynamic stress, with increased heart rate, blood volume, and blood pressure [31]. However, recent recognition of the inflammatory state present in obesity, believed to be in large part mediated by adipocyte secretory products and which may underlie insulin resistance [32], has changed our understanding of the pathophysiologic links of obesity with diabetes and cardiovascular diseases [32]. Moreover, many of these immune-inflammatory mediators may have important roles in ventricular remodeling [33] and heart failure [34]. The frequently large associations seen here with adiposity indices and the important reduction in diabetes associations seen with adjustment for these indices provides epidemiologic evidence supporting the hypothesis that these processes are indeed active in the pathophysiology of ventricular dysfunction.

There are, however, additional specific mechanisms by which diabetes might be related to LV geometry and hypertrophy. Diabetes may contribute to ventricular adaptive changes through an increase in pro-inflammatory immune mediators resulting from the oxidative stress caused by hyperglycemia [32,35]. The association may also be mediated through the production of advanced glycation end products (AGE), which may, by leading to a reduced degradation of collagen, impair ventricular compliance [36].

Some limitations to our study merit mention. Forty percent of the initially enrolled participants could not be analyzed, creating the possibility of selection bias influencing our results. In this regard, as those included were generally healthier than those excluded, and the associations we have demonstrated may underestimate the true association between diabetes and LVH. The high prevalence of obesity makes these results of questionable generalizability to leaner populations. Finally, the high prevalence of examined echocardiographic features results in the reported odds ratios being overestimates of relative risk.

## Conclusion

In conclusion, diabetes is associated with increased LVH in middle-aged and elderly African-American men and women. Most of this increase appears to be more closely associated with obesity, especially central obesity, than to diabetes *per se*. Diabetes associated altered ventricular structure appears to differ by gender. This may be due to gender difference in the cardiac response to the pathophysiologic factors underlying LV morphology – in men the process of increased mass being more coupled with chamber dilation whereas in women expressed mostly as wall thickening. The gender difference in LV structure complicates the use of common geometric patterns definitions when comparing ventricular remodeling in response to diabetes.

## Competing interests

The author(s) declare that they have no competing interests.

## Authors' contributions

MF and BBD designed and performed the statistical analysis and drafted the manuscript. All authors helped with suggestions in analysis and manuscript, read and approved the final manuscript.
